# Supplementary material for: Resistance to MET/VEGFR2 Inhibition by Cabozantinib Is Mediated by YAP/TBX5-Dependent Induction of FGFR1 in Castration-Resistant Prostate Cancer
Source: Cancers (Basel). 2020 Jan 19;12(1):244. doi: 10.3390/cancers12010244 (PMC7016532; doi:10.3390/cancers12010244)
Supplement: Supplementary file 1 [file cancers-12-00244-s001.pdf]

Supplementary Materials

Resistance to MET/VEGFR2 inhibition by cabozantinib is mediated by YAP/TBX5-dependent induction of FGFR1 in castration-resistant prostate cancer

Filippos Koinis, Paul Corn, Nila Parikh, Jian Song, Ioulia Vardaki, Ioanna Mourkioti, Sue-Hwa Lin, Christopher Logothetis, Theocharis Panaretakis and Gary Gallick

Table S1. shRNA sequences

Met1 shRNA#1 target	GTAAAGAGGCACTAGCAAA
Non-Target control shRNA	TTCTCCGAACGTGTCACGT
TBX5 shRNA#1 target	GCCGACGATCACAGATACAAA
TBX5 shRNA#2 target	GCTGCACAGAATGTCAAGAAT
YAP shRNA#1 target	GCCACCAAGCTAGATAAAGAA
YAP shRNA#2 target	CAGGTGATACTATCAACCAAA
Non-target Control shRNA (RFP)	SIGMA-ALDRICH, Lot 03241711MN
PCDNA3.1-FGFR1+P2A-eGFP	GeneScript, Clone ID NM_023110.2

Table S2. PCR primers

GAPDH	Forward Primer	5′-CCTGCACCACCAACTGCTTA-3′
	Reverse Primer	5′-GGCCATCCACAGTCTTCTGAG-3′
FGF1	Forward Primer	5′-GGGCTTTTATACGGCTCACA-3′
	Reverse Primer	5′-TGCTTCTTGGATATATAGGTGTTGTAA-3′
FGF2	Forward Primer	5′-AGAAGAGCGACCCTCACATCA-3′
	Reverse Primer	5′-CGGTTAGCACACACTCCTTTG-3′
TBX5	Forward Primer	5′-GGGCTCCCAGTACCAGTGTGA-3′
	Reverse Primer	5′-GTAGGGCTTCTTGTAGGGATG-3′
GINS1	Forward Primer	5′-CCCCAAAAGTGTCCATGACC-3′
	Reverse Primer	5′-CAGGATCACCCCCATCTCAA-3′
RRM2	Forward Primer	5′-CTGTTTCTATGGCTTCCAAAT-3′
	Reverse Primer	5′-TTCTTCTTCACACAAGGCATT-3′
YAP	Forward Primer	5′-CGCTCTTCAACGCCGTCA-3′
	Reverse Primer	5′-AGTACTGGCCTGTCGGGAGT-3′

Table S3. Antibodies used in the study.

**Antigent**	**Dilution**	**Catalogue No**	**Source**
		**Immunohistochemistry**	
FGFR-1	1:300	NB100-2080	Novus
TBX5	1:500	PA5-29845	Invitrogen
YAP	1:500	12395	Cell signaling
CD31	1:100	77699	Cell signaling
		**Immunofluorecence**	
TBX5 (A-6)	1:100	sc-515536	Santa Cruz
YAP	1:250	NBP110-58358	Novus Biologicals, CO
		**Immunoblotting**	
MET (D1C2)	1:1000	8198	Cell Signaling

p-MET (Y1234/1235)	1:1000	3077	Cell Signaling
YAP (1A12)	1:1000	12395	Cell Signaling
p-YAP (S127) (D9W2I)	1:1000	13008	Cell Signaling
FGFR-1 (D8E4)	1:1000	9740	Cell Signaling
p-FGFR-1 (T766) (1E5)	1:1000	2544	Cell Signaling
TBX5 (A-6)	1:1000	sc-515536	Santa Cruz
β-actin (13E5)	1:1000	4970	Cell Signaling
Vinculin	1:10000	V9131	Sigma-Aldrich, St. Luis, MO, USA
AR (N-20)	1:1000	sc-816	Santa Cruz
p-LATS1 (S909)	1:1000	9157	Cell Signaling
LATS1 (C66B5)	1:1000	3477	Cell Signaling
MST1	1:1000	3682	Cell Signaling
p-MST1/2	1:1000	3681	Cell Signaling

Figure 1D

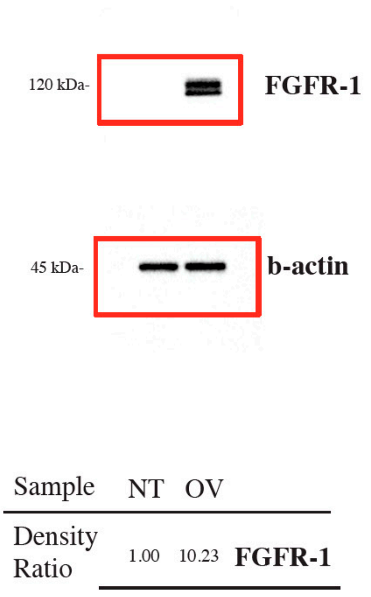

Figure 1G

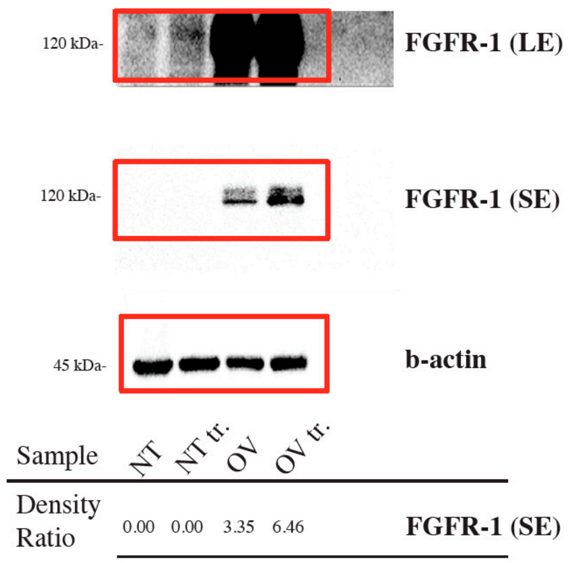

Figure 2C

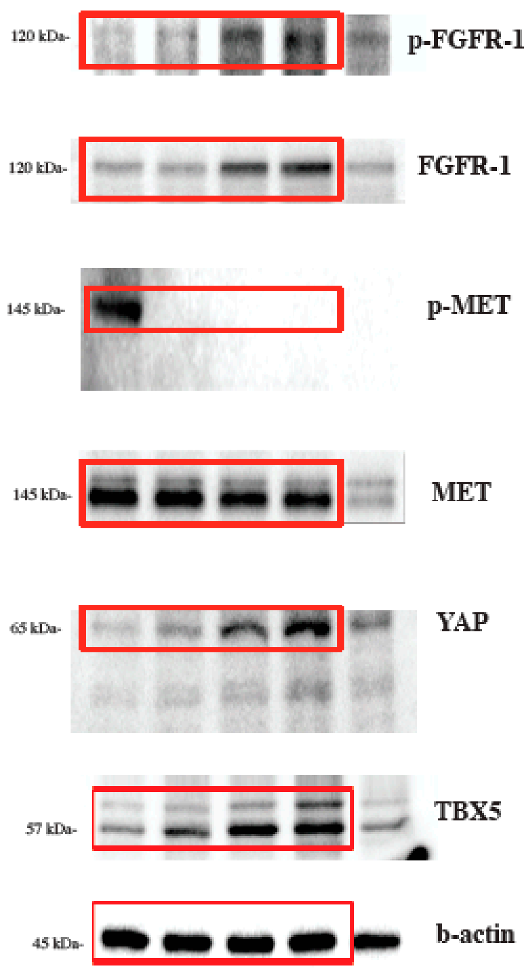

	Cabo (1uM)				
Sample	0	14	28	42	Days
Density Ratio	1.00	1.89	6.59	8.47	p-FGFR-1
	1.00	1.08	3.06	4.32	FGFR-1
	1.00	0.00	0.00	0.00	p-MET
	1.00	0.86	0.78	0.80	MET
	1.00	2.20	4.32	9.33	YAP
	1.00	3.12	4.84	7.31	TBX5

Figure 2D

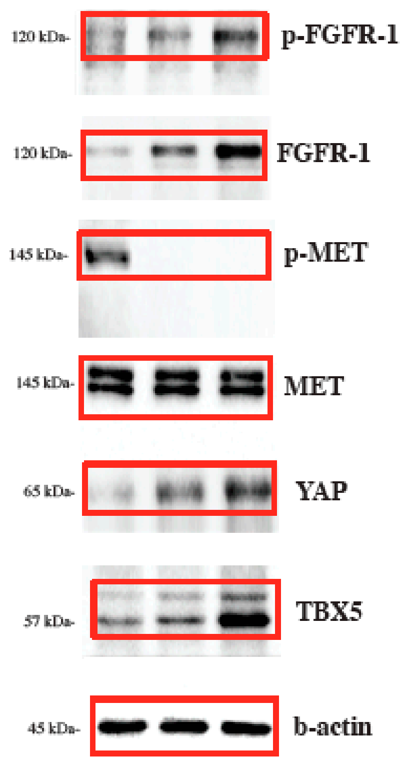

Sample	0	0.8	1	Cabo (1uM)
Density Ratio	1.00	1.55	4.05	p-FGFR-1
	1.00	3.25	5.12	FGFR-1
	1.00	0.00	0.00	p-MET
	1.00	1.03	0.99	MET
	1.00	5.11	7.83	YAP
	1.00	2.35	7.19	TBX5

Figure 2E

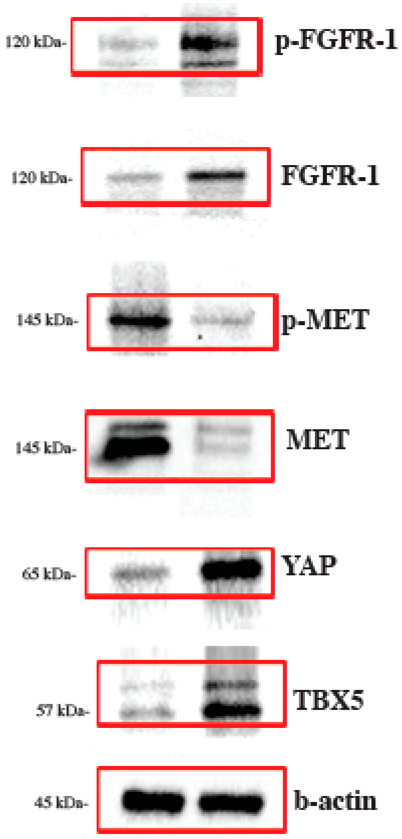

Sample	NT	shMET	
Density Ratio	1.00	5.49	**p-FGFR-1**
	1.00	5.71	**FGFR-1**
	1.00	0.27	**p-MET**
	1.00	0.18	**MET**
	1.00	9.81	**YAP**
	1.00	8.12	**TBX5**

Figure 3A

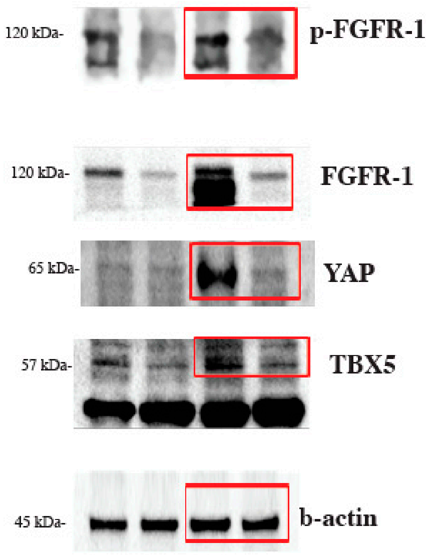

Sample	PC3	144-13	
Density Ratio	1.00	0.35	p-FGFR-1
	1.00	0.11	FGFR-1
	1.00	0.17	YAP
	1.00	0.35	TBX5

Figure 3D

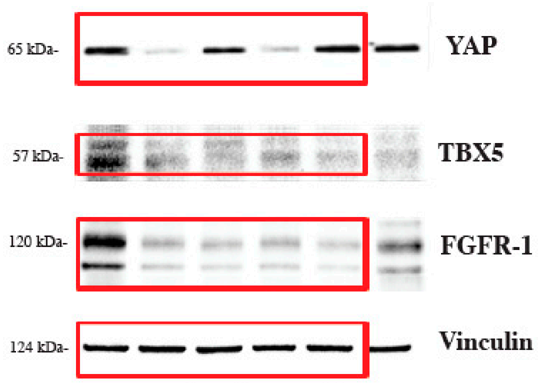

Sample	NT	shYAP-1	shTBX5-1	shYAP-2	shTBX5-2	
Density Ratio	1.00	0.10	0.85	0.18	0.90	YAP
	1.00	0.23	0.18	0.22	0.06	TBX5
	1.00	0.18	0.13	0.19	0.10	FGFR-1

Figure 4B

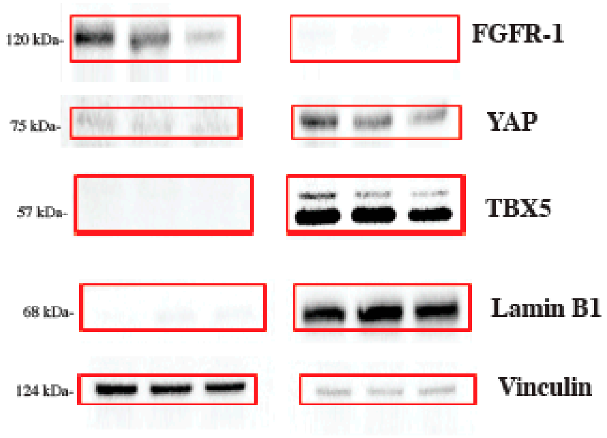

	Cytoplasm			Nucleus			
Sample	0	24	48	0	24	48	
Density Ratio	1.00	0.83	0.27	0.05	0.02	0.01	FGFR-1
	0.15	0.09	0.08	1.15	0.77	0.69	YAP
	0.22	0.17	0.10	1.15	0.84	0.81	TBX5

*Cytoplasmic extracts were normalzed to vinculin, whereas nucleic to Lamin B1.

Figure 4C

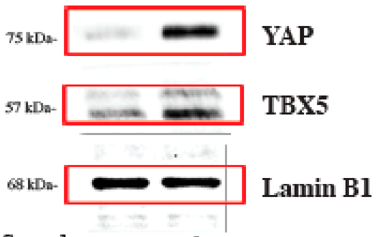

Sample	0	1	Cabo (uM)
Density Ratio	1.00	6.31	YAP
	1.00	2.77	TBX5

Figure 4D

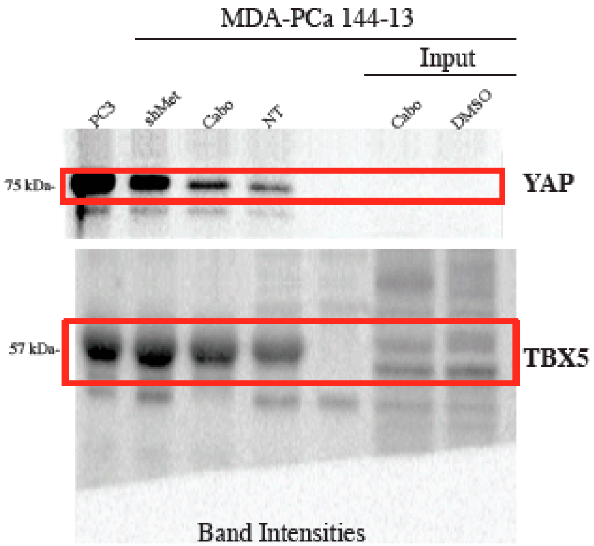

Band Intensities

1.00	0.71	0.44	0.34	0.00	0.00	YAP
1.00	1.12	0.94	0.74	0.65	0.65	TBX5

Figure 4E

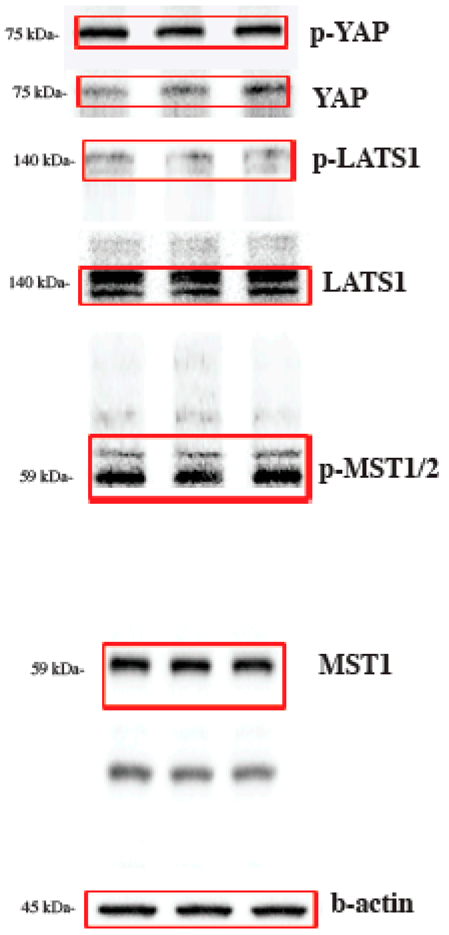

Sample	0	0.8	1	Cabo (uM)
Density Ratio	1.00	0.91	0.98	p-YAP
	1.00	1.37	2.09	YAP
	1.00	1.30	1.27	p-LATS1
	1.00	0.93	1.02	LATS1
	1.00	0.96	1.10	p-MST1/2
	1.00	1.00	0.99	MST1/2

Figure 5A

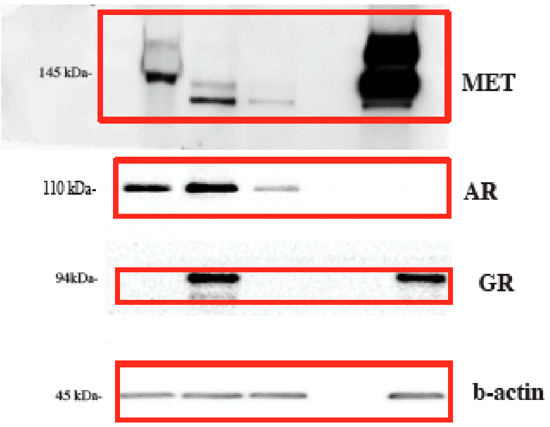

Sample	905L	LREX	LNCaP	PC3	
Density Ratio	9.75	6.61	3.33	19.49	MET
	3.25	4.74	1.10	0.00	AR
	0.01	3.77	0.00	3.11	GR

Figure 5B

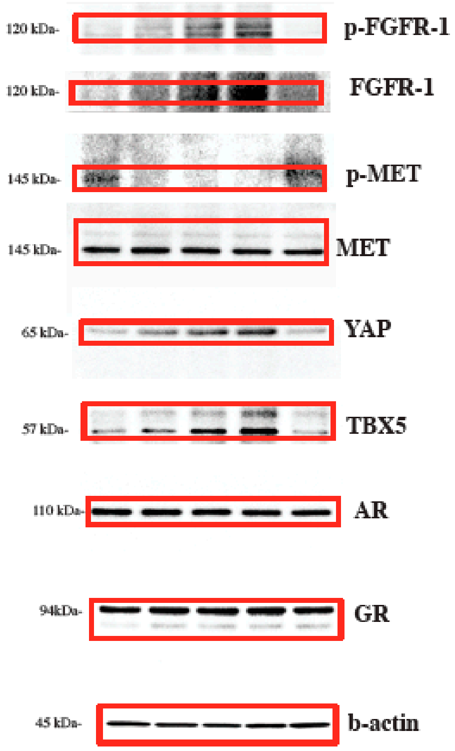

	Cabo (1uM)				DMSO	
Sample	0	14	21	28	28	Days
Density Ratio	1.00	1.83	4.45	7.57	0.74	p-FGFR-1
	1.00	3.20	7.66	12.13	2.14	FGFR-1
	1.00	0.18	0.14	0.06	1.12	p-MET
	1.00	1.34	1.48	1.17	1.02	MET
	1.00	3.12	5.73	7.18	1.02	YAP
	1.00	2.81	8.16	12.56	1.02	TBX5
	1.00	1.14	1.26	1.00	1.84	AR
	1.00	1.33	1.59	1.36	1.04	GR

Figure 5C

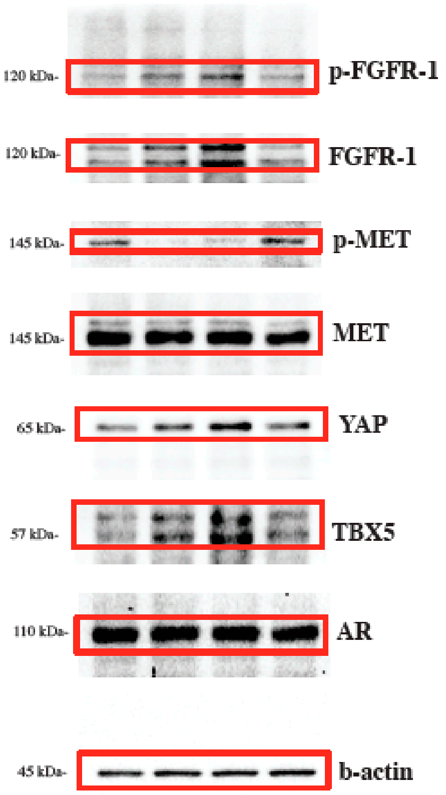

LNCaP

	Cabo (1uM)			DMSO	
Sample	0	14	21	21	Days
Density Ratio	1.00	1.83	3.20	0.89	p-FGFR-1
	1.00	3.90	7.78	1.00	FGFR-1
	1.00	0.04	0.27	0.95	p-MET
	1.00	0.80	0.82	0.73	MET
	1.00	2.85	7.32	1.10	YAP
	1.00	2.63	5.82	1.00	TBX5
	1.00	0.85	0.86	0.74	AR

Figure 5D

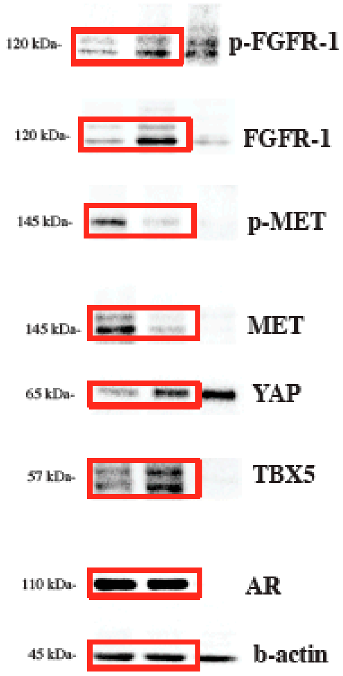

LREX

Sample	NT	shMet	
Density Ratio	1.00	3.54	p-FGFR-1
	1.00	5.64	FGFR-1
	1.00	0.10	p-MET
	1.00	0.20	MET
	1.00	3.24	YAP
	1.00	4.83	TBX5
	1.00	0.96	AR

© 2020 by the authors. Licensee MDPI, Basel, Switzerland. This article is an open access article distributed under the terms and conditions of the Creative Commons Attribution (CC BY) license (http://creativecommons.org/licenses/by/4.0/).
